# Parthenogenetic bovine embryos secrete type I interferon capable of stimulating *ISG15* in luteal cell culture

**DOI:** 10.21451/1984-3143-AR2018-0095

**Published:** 2018-12-05

**Authors:** Alessandra Bridi, Kalyne Bertolin, Vitor B. Rissi, Lady K.S. Mujica, Werner G. Glanzner, Mariana P. de Macedo, Fabio V. Comim, Paulo B.D. Gonçalves, Alfredo Q. Antoniazzi

**Affiliations:** Laboratory of Biotechnology and Animal Reproduction – BioRep, Department of Large Animal Clinical Science, Federal University of Santa Maria, Santa Maria, RS, Brazil.

**Keywords:** ISG15, IFNT, luteal cell culture, IFNA, bovine

## Abstract

Interferon tau (IFNT) is the pregnancy recognition signal in ruminants and is secreted by trophoblast cells. Paracrine action in the endometrium is well established by inhibiting luteolytic pulses of prostaglandin F2 alpha. Recently, endocrine action was documented in the corpus luteum, blood cell and liver. It was hypothesized that conditioned medium (CM) obtained from days 7, 9 and 12 parthenogenetic embryos alters luteal cell gene expression. The aim was to establish a bovine mixed luteal cell culture to evaluate cellular response associated to interferon stimulated genes, steroidogenesis and apoptosis. Conditioned medium was obtained from Days 7, 9 and 12 parthenogenetic (PA) embryos culture. Moreover, antiviral assay was performed on CM from Days 7, 9 and 12 to verify Type I interferon activity. Luteal cell culture was validated by steroidogenic and apoptotic genes (*CYP11A1* , *HSD3B1, BAX*, *BCL2*, *AKT* and *XIAP* mRNA expression), and concentration of progesterone as endpoint. Luteal cell culture was treated with interferon alpha (IFNA) and CM from parthenogenetic embryos. Antiviral assay revealed Type I interferon activity on CM from embryos increasing on Days 9 and 12. *ISG15* mRNA was greater in the mixed luteal cells culture treated with 1, 10 and 100ng/ml of interferon alpha (IFNA) and also on Days 7, 9 and 12 CM treatments. Concentration of progesterone was not altered in luteal cell culture regardless of treatments. Steroidogenic and apoptotic genes were similar among groups in luteal cell culture treated with different doses of IFNA or CM from PA embryos. In conclusion, parthenogenetic embryo-derived CM has antiviral activity, luteal cell culture respond to Type I interferon by expressing IGS15. These data indicate this model can be used for IFNT endocrine signaling studies.

## Introduction

Embryonic losses in early pregnancy result in premature return to estrus on dairy and beef cattle. In bovine, around 40% of embryo mortality occurs between Days 8 and 17 of pregnancy ( Thatcher *et al*., 2001 ), corresponding to the period of interferon tau (IFNT) secretion by the trophectoderm, which characterizes the pregnancy recognition signal in ruminants ( Imakawa *et al*., 1987 ). IFNT is a cytokine and acts in a paracrine manner inside the uterus by decreasing the expression of estrogen and oxytocin receptors in the uterine endometrium, and inhibiting the endometrial pulses of prostaglandin F2 alpha (PGF) to avoid luteolysis ( Roberts *et al*., 2008 ). In addition to its antiluteolytic effects, IFNT increases the expression of interferon-stimulated genes (ISGs) in the endometrium ( Austin *et al*., 2004 ), and in other extra uterine tissues such as blood ( Han *et al*., 2006 ), liver and corpus luteum (CL) ( Oliveira *et al*., 2008 ).

Recent studies support the hypothesis that the signal of maternal recognition of pregnancy also occurs in an endocrine manner in ruminants. In ewes, IFNT secreted by the conceptus reaches the uterine vein, inducing ISGs in the blood cells at early stages of pregnancy ( Gifford *et al*., 2007 ; Oliveira *et al*., 2008 ; Bott *et al*., 2010 ). IFNT acts on extra uterine tissues through systemic circulation, reaching the CL and stimulating ISGs expression in luteal cells ( Oliveira *et al*., 2008 ; Bott *et al*., 2010 ). Interferon-stimulated gene 15 (ISG15) is stimulated by Type 1 interferon (IFN), also called ubiquitin cross-reactive protein ( Haas *et al*., 1987 ), and it is upregulated in endometrial cells of the mouse ( Austin *et al*., 2003 ), human ( Bebington *et al*., 1999 ) and ruminants ( Austin *et al*., 1996 ) during early pregnancy. For this reason, ISG15 can be used as an indirect evidence of IFNT-dependent cellular response. IFNT binds to Type 1 interferon receptor (IFNAR1 and IFNAR2) to induce a response, and conjugates to intracellular proteins ( Loeb and Haas, 1992 ) in a mechanism similar to ubiquitin ( Narasimhan *et al*., 1996 ).

In ovine, ISG15 mRNA expression in large luteal cells is upregulated on Day 15 pregnant when compared to non-pregnant ewes. Moreover, IFNT concentrations were greater in uterine artery, uterine vein and jugular vein blood on Day 15 pregnant ewes ( Oliveira *et al*., 2008 ). In another experiment, serum progesterone concentrations were maintained after PGF injection in IFNT-infused non-pregnant ewes for 24h ( Antoniazzi *et al*., 2013 ). Therefore, strong and recent evidences indicate that IFNT acting in an endocrine manner has an essential role during the initial period of pregnancy recognition in ruminants.

Studies involving IFNT signaling on extra uterine tissues started about ten years ago, and have been focused on *in vivo* models, whereas only few studies used *in vitro* models for this purpose. For this reason, our hypothesis is that conditioned medium (CM) from bovine parthenogenetic (PA) embryos have Type I IFN activity capable of modulating *ISG15* mRNA expression in luteal cell culture. Consequently, the aim of this study was to establish a bovine luteal cell culture to evaluate its response when treated with CM on interferon stimulated genes (*ISG15*), steroidogenic (*CYP11A1 *, *HSD3B*) and apoptotic (*BAX*, *BCL2 *, *AKT* and *XIAP*) gene expression. Furthermore, this may be a potential method for studying endocrine signaling of IFNT

## Materials and Methods

All experimental procedures used bovine ovaries from slaughterhouse, and were approved by the Animal Ethics and Use Committees (CEUA nº 1563271115) at Federal University of Santa Maria.

### Oocyte retrieval and parthenogenetic activation

Bovine ovaries, for embryo production, were obtained from the slaughterhouse and transported to the laboratory in a 0.9% NaCl solution containing penicillin (100IU /ml) and streptomycin sulfate (50 μg/ml) at 32°C. Oocytes were aspirated from follicles 3-8 mm in diameter, and cultured for 22 to 24h, under 5% CO_2_ in air at 38.5°C, using TCM-199 (Gibco Labs, Waltham, MA, USA) supplemented with 10% fetal bovine serum (FBS), 5.0 mg/ml Luteinizing Hormone (LH), 0.5mg/ml Follicle Stimulating Hormone (FSH) (Folltropin ^®^-V, Bioniche, Ontario, CA, USA), 0.2mM pyruvate, 100 IU/ml of penicillin and 50μg/ml of streptomycin. After *in vitro* maturation (IVM), oocytes were denuded by vortexing with hyaluronidase (0.1%). Oocytes were washed four times in TCM-199, with 0.2% bovine serum albumin (BSA) and 100I U/ml of penicillin and 50 μg/ml of streptomycin before and after activation treatment. For the oocyte activation protocol, 5 µM of ionomycin for 5 min was used. Then, oocytes were washed one more time and cultured in 2 mM 6-dimethylaminopurine for 4 h ( Méo *et al*., 2007 ). Finally, parthenogenetically activated oocytes were cultured in modified synthetic oviduct fluid (SOF) supplemented with 5% FBS, 100 IU/ml of penicillin and 50 μg/ml of streptomycin under 5% CO_2_, 5% O_2_ and, 90% N_2_ at 38.5°C, up to Days 7, 9 or 12, when the conditioned medium (CM) was stored at -80°C for subsequent usage. Cleavage was assessed 48 h after activation and the average rate was 79.6%. Blastocyst development was observed on Day 7 after activation and the rates were about 38% over cleaved. The average number of embryos per group was 17 on Day 7, 14 on Day 9 and 3 embryos on Day 12.

### Corpus Luteum Dissociation and Cell Preparation

Bovine ovaries were obtained from the slaughterhouse and transported to the laboratory in phosphate-buffered saline (PBS) containing penicillin (100 IU/ml) and streptomycin sulfate (50 μg/ml) at 4ºC for corpus luteum *in vitro* culture. The CLs were classified as early (1-6 days post-ovulation), mid (8-12 days post-ovulation), and late (15-17 days post-ovulation) according to the criteria established by Miyamoto ( Miyamoto *et al*., 2000 ). For this experiment, CLs were selected to be in early or mid estrous cycle, 15-25 mm in diameter; presenting luteal tissue on the ovarian surface; the color blood to pink, tan or orange; and compact soft consistency.

Luteal tissue was mechanically separated from its fibrous capsule, chopped into small pieces with scalpel blade, and further dissociated in a 0.1% collagenase Type IA (Sigma-Aldrich, St Louis, MO, USA), and DMEM-F12 solution for 30 min at 37°C. After, collagenase was inactivated with DMEM-F12 solution supplemented with 10% FBS, 1% antibiotics (penicillin 10.000 IU/ml and streptomycin 10.000 µg/ml). Cell suspension was washed and centrifuged twice for 10 min at 200 Xg with DMEM-F12 solution supplemented with 10% FBS, 1% antibiotics. Then, luteal cells were plated on 60 mm dishes for 48 h at 37°C in 5% CO_2_. After 48 h culture, the medium was changed. The viability of the luteal cells was assessed by the Trypan Blue (Sigma-Aldrich, St Louis, MO, USA) when the cells were trypsinized, and then they were seeded again in 96-well plates at 3 x 10^4^ cells/well. For cell culture adaptation, luteal cells were cultured in SOF supplemented with 5% FBS, 100 IU/ml of penicillin and 50 μg/ml of streptomycin for 12 h. Next, the cells received treatments as detailed in the experimental design 1 and 2.

### Concentrations of progesterone in luteal cell culture media

Concentration of progesterone was determined by chemiluminescence kit (ADVIA Centaur, Siemens). The sensitivity of the assay was 0.15 ng/ml. Progesterone concentration was measured on CM from luteal cell culture following experimental design.

### RNA extraction, reverse transcription and real-time PCR

Extraction of RNA from luteal cell culture was performed using TRIzol®. Quantification and estimation of RNA purity was performed using a Nano-Drop spectrophotometer (Thermo Scientific – Waltham USA; Absorbance 260/280 nm ratio). RNA was treated with 0.1 U DNase Amplification Grade (Invitrogen) for 15 min at 27^o^C, to digest any contaminating DNA. After, DNase was inactivated by adding 1 ul of EDTA at 65^o^C for 10 min. Single-stranded cDNA was synthesized from 1000 ng of total RNA with final volume of 20 ul using iScript cDNA Synthesis Kit (BioRad, Hercules, CA) according to the manufacturer’s instructions. Quantitative polymerase chain reactions (qPCR) were conducted in a CFX384 thermocycler (BioRad) using a final volume of 10 ul per well using BRYT Green® dye and Taq DNA polymerase from GoTaq® qPCR Master Mix (Promega Corporation), with cDNA (2 ul) and bovine-specific primers ( Tab. 1 ). Amplification was performed with initial denaturation at 95^o^C for 5 min followed by 40 cycles of denaturation at 95^o^C for 15 sec and annealing/extension at 60^ o^C for 30 sec.

**Table 1:** Real time qPCR primer sequences.

Target	Accession	Primers sequences
*GAPDH*	NM_001034034.2	F tgaccccttcattgaccttc R cgttctctgccttgactgtg
*RPL19*	NM_001040516.1	F ccggctgcttagacgatacc R ccgcttgtttttgaacacgtt
*ISG15*	NM_174366	F ggtatccgagctgaagcagtt R acctccctgctgtcaaggt
*BAX*	NM_173894	F ttctgacggcaacttcaact R cgaaggaagtccaatgtcca
*BCL2*	NM_001166486.1	F cctatctgggccataagtgaag R gtggtgcatcagcaacaatg
*AKT*	NM_173986.2	F gattcttcgccagcatcgtg R ggccgtgaactcctcatcaa
*XIAP*	NM_001205592.1	F gaagcacggatcattacatttgg R ttcacctaaagcataaaatccag
*CYP11A1*	NM_176644.2	F cttgcacctttctggctagg R aaggggaagaggtagggtga
*HSD3B1*	NM_174343.3	F gcccaactcctacagggagat R ttcagagcccacccattagct

F, forward; R, reverse.

To optimize the qPCR assay, serial dilutions of cDNA templates were used to generate a standard curve. The standard curve was constructed by plotting the log of the starting quantity of the dilution factor against the Ct value obtained during amplification of each dilution. Reactions with a coefficient of determination (R2) higher than 0.98 and efficiency between 85 to 110% were considered optimized. The relative standard curve method was used to assess the amount of a particular transcript in each sample. Samples were run in duplicate and results are expressed relative to GAPDH and RPL19. The target genes are presented on Table1 .

### Interferon-induced antiviral activity assay

Interferon-induced antiviral activity of the samples were determined based on a modified antiviral assay ( Vogel *et al*., 2001 ). Madin-Darby bovine kidney cells (MDBK) were used for amplification and titration of vesicular stomatitis virus, and for IFN-induced antiviral activity assay. Briefly, samples and IFN control (100 IU of IFNA) were diluted in base 2 (4 to 2048) in 96-well plate and a suspension of MDBK cells was added. The plate was maintained in CO_2_ atmosphere for 12-18 h, at 37°C. Vesicular stomatitis virus were inoculated at 100 tissue culture infectious doses (TCID_50_) in all dilutions of the samples, IFN and virus control. The dishes were maintained in CO_2_ atmosphere_,_ for 24 h, at 37°C. The first well in the dilution series of a sample that exhibits cytopathic effect equivalent to that of the virus control wells was defined as the endpoint. Title samples were established based on IFNA control.

### Experimental design

#### Experiment 1: Validation of bovine luteal cell culture viability and IFNA responsiveness

This study evaluated the dose-dependent response in luteal cell culture treated with IFNA. Relative *ISG15* mRNA expression, steroidogenic genes (*CYP11A1 *, *HSD3B1*), and apoptosis related genes (*BAX, BCL2, AKT* and *XIAP*) were assessed in order to validate this model. For this, luteal cells were treated *in vitro* for 24h with 0 (SOF), 0.1, 1, 10, or 100 ng/ml of IFNA (IFN 2 alpha – ROCHE). Following treatment, cells were washed with PBS and collected using TRIzol^®^, and stored at -80°C for subsequent RNA extraction. In the culture medium, P4 concentration was measured in control, and on groups treated with 1 and 100ng/ml of IFNA. The experiment was repeated four times, and each replicate used 5 different CLs as previously stated.

#### Experiment 2: Exposure of bovine luteal cell to conditioned medium derived from parthenogenetic embryos

After validating luteal cell culture responsiveness to IFNA, the aim of the second experiment was to verify whether mixed luteal cell culture respond similarly to CM from parthenogenetic activated embryos cultured for 7, 9 or 12 Days. Luteal cells were treated for 24 h with SOF (control; without embryos) and conditioned medium from parthenogenetic activated embryos (CM) collected on Days 7, 9 and 12 of the embryonic development. Subsequently, luteal cells were collected for RNA extraction as previously described. Interferon-induced antiviral activity was assessed in CM from embryos on Days 7, 9 and 12 to evaluate the bioactivity of Type I IFN. Concentration of progesterone was measured in pools of CM from embryos on days 7, 9 and 12 after 24 h of treatment. The experiment was performed in three replicates of parthenogenetic activated embryos and two replicates of luteal cell culture, (n = 5 CLs per replicate).

### Statistical analysis

All data were analyzed using JMP (Version 7.0 SAS Institute Inc.) statistical software. The mRNA expression data were analyzed using ANOVA and differences between treated and control luteal cell culture were compared by Tukey’s test. All numerical data are represented as mean ± SEM. Significant differences were considered at P ≤ 0.05.

## Results

### Viability of mixed luteal cells cultured in vitro and progesterone secretion in the culture medium

The viability of luteal cells ranged from 80% to 90% as evaluated by the Trypan Blue method for both IFNA and CM treated cells (data not shown). Concentration of progesterone was determined in the culture medium 24 h after luteal cells treatment with IFNA or CM ( Tab. 2 ). IFNA and CM have no impact on progesterone productions. These data indicate that luteal cell cultured *in vitro* produce P4, validating the system. However, P4 production is not influenced by IFNA or CM.

**Table 2:** Concentration of progesterone produced by luteal cells in culture at 24 h of treatment with IFNA or conditioned medium (CM) from parthenogenetic embryos.

Luteal cells treatment	P4 concentration (ng/ml)
SOF (positive control)	0.21
PA embryo CM 7, 9 and 12 after 24 h treatment –1º replicate	181.32
PA embryo CM 7, 9 and 12 after 24 h treatment – 2º replicate	132.06
Culture medium after 24 h treatment (control IFNA)	145
Culture medium after 24 h treatment (1 ng/ml IFNA)	192.60
Culture medium after 24 h treatment (100 ng/ml IFNA)	184.12

### Antiviral assay of Type I IFN activity

**Figure 1:**
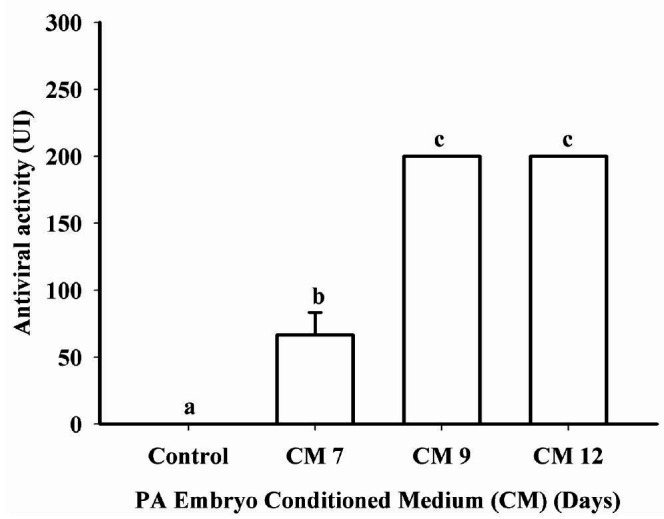
Antiviral activity (nontransformed data) detected in conditioned medium (CM) from parthenogenetic activated (PA) bovine embryos on Days 7, 9 or 12 of development. Antiviral activity from CM was detected on Days 7, 9 or 12 when compared to control (medium with no embryos). The levels of Type I IFN were 50 to >200 UI/ml in the CM. Data are presented as mean ± SEM and differences (P < 0.05) are represented by different lowercase letters.

Conditioned medium presented antiviral activity compatible with Type I IFNs on Days 7, 9 and 12. Antiviral activity in the CM of PA embryos on Days 9 and 12 was greater (P < 0.0001) than on Day 7 and control groups. The levels of Type I IFN activity ranged from 50 to >200 UI/ml on CM ( Fig. 1 ).

### Interferon stimulated gene 15 (ISG15) mRNA expression in mixed luteal cell exposed to IFNA or CM

**Figure 2:**
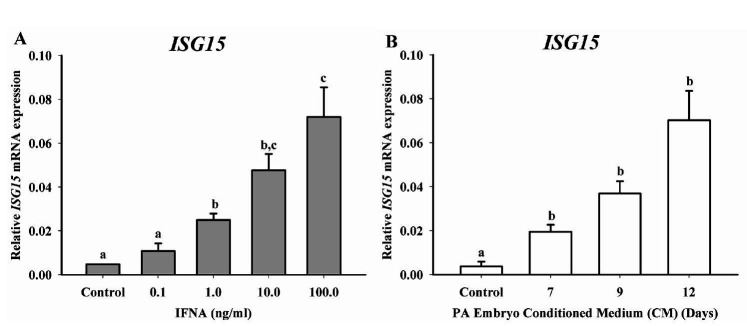
*ISG15* mRNA expression in luteal cell culture following treatment with different concentrations of IFNA or conditioned medium (CM) from parthenogenetic activated (PA) embryos on Days 7, 9 or 12. (A) Relative *ISG15* mRNA expression in luteal cells treated with control (medium with no embryos), 0.1, 1, 10 or 100 ng/ml of IFNA. Luteal cells increased (P < 0.0001) *ISG15* mRNA expression in a dose-dependent manner, responding to 1 ng/ml. (B) Relative *ISG15* mRNA expression in luteal cell culture after treatment with CM from PA embryos on Days 7, 9 or 12. *ISG15* mRNA expression on Days 7, 9 and 12 were greater (P = 0.0001) when compared to control. Data are presented as mean ± SEM and differences (P < 0.05) are indicated by different lowercase letters.

Relative *ISG15* mRNA expression was evaluated on luteal cells culture treated for 24 h with different doses of IFNA (control, 0.1, 1, 10 or 100 ng/ml) or CM on Days 7, 9 or 12. Luteal cells responded to 1ng/ml of IFNA increasing *ISG15* mRNA expression (P < 0.0001), at 10 and 100 ng/ml when compared to control ( Fig. 2A ). Subsequently, luteal cells treatment with CM from different embryonic developmental days (7, 9 and 12), increased (P = 0.0001) *ISG15* mRNA expression when compared to control group ( Fig. 2B ). Considering IFNA as positive control, both treatments (IFNA and CM) presented a similar *ISG15* mRNA expression. These data indicate that luteal cells culture treated with CM from PA embryos can induce *ISG15* in a developmental dependent manner.

### Expression pattern of steroidogenic and cell survival genes by bovine luteal cells in response to IFNA or conditioned medium derived from parthenogenetic embryos

**Figure 3:**
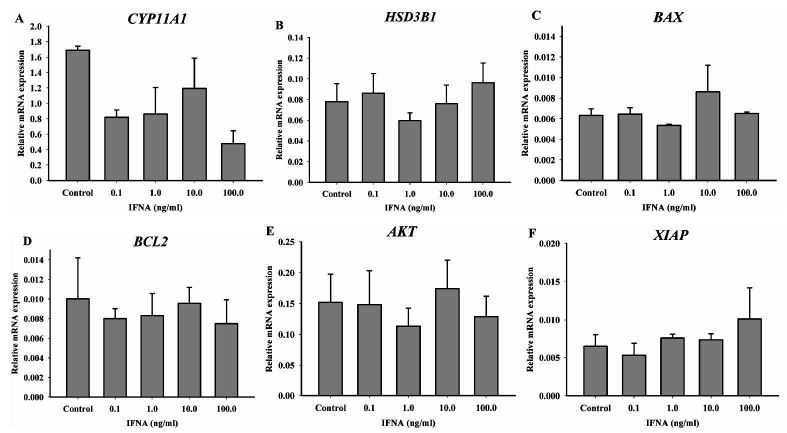
Steroidogenesis and cell survival genes expression on IFNA treated luteal cell culture. Luteal cells mRNA expression of steroidogenic enzymes (A) *CYP11A1* , (B) *HSD3B1* and cell survival genes (C) *BAX*, (D) *BCL2*, (E) *AKT* and (F) *XIAP* following 24 h treatment with different doses of IFNA (0, 0.1, 1, 10 or 100 ng/ml). *CYP11A* and *HSD3B1* mRNA expression were not different. Cell survival genes were not affected by different concentrations of IFNA on luteal cell culture. Data are presented as mean ± SEM and differences are represented by different lowercase letters.

Luteal cell culture was treated for 24 h with different doses of IFNA (control, 0.1, 1, 10 or 100 ng/ml) to evaluate the expression of steroidogenic genes (*CYP11A1* , *HSD3B1*) and cell survival genes (*BAX*, *BCL2 *, *AKT* and *XIAP*). The expression of *CYP11A1* ( Fig. 3A ) and *HSD3B1* ( Fig. 3B ) was not different regardless of IFNA dose, indicating that IFNA does not regulate the transcription of steroidogenic genes, complementing the progesterone production data. Apoptosis/survival genes *BAX*, *BCL2*, *AKT* and *XIAP* mRNA were not affected by different concentrations of IFNA in luteal cell culture ( Fig. 3C-F ), These data suggest IFNA does not regulate the transcription of genes associated with cell survival, confirming luteal cell viability.

**Figure 4:**
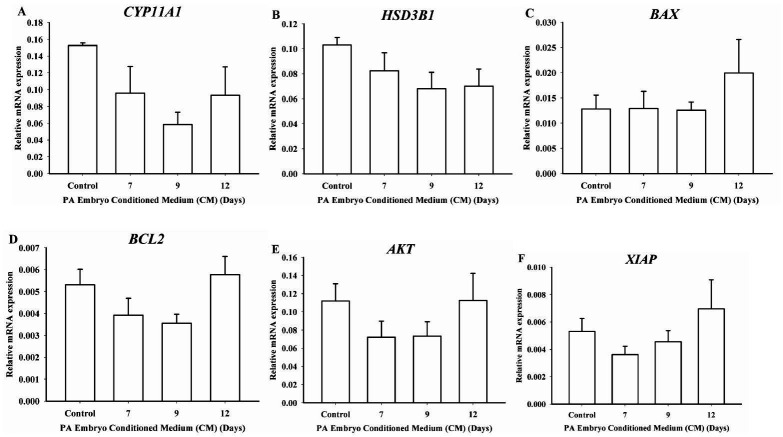
Steroidogenesis and cell survival genes expression on luteal cell culture treated with conditioned medium (CM) from parthenogenetic activated (PA) embryos. Luteal cells mRNA expression of steroidogenic enzymes (A) *CYP11A1*, (B) *HSD3B1* and cell survival genes (C) *BAX*, (D) *BCL2 *, (E) *AKT* and (F) *XIAP* following 24 h treatment with CM from PA embryos on Days 7, 9 or 12. Luteal cell culture treated with CM did not change mRNA expression encoding for steroidogenic enzymes. Cell survival genes presented no differences among groups. Data are presented as mean ± SEM and differences are represented by different lowercase letters.

Relative mRNA expression of steroidogenic enzymes (*CYP11A1*, *HSD3B1*) and cell survival genes (*BAX*, *BCL2* , *AKT* and *XIAP*) were assessed on luteal cell culture treated for 24h with CM from Days 7, 9 or 12. *CYP11A1* ( Fig. 4A ) and *HSD3B1* ( Fig. 4B ) mRNA expression did not vary regardless of day of embryo culture when CM was obtained. For the cell survival genes, *BAX* ( Fig. 4C ), *BCL2* ( Fig. 4D ), *AKT* ( Fig. 4E ) and *XIAP* ( Fig. 4F ) mRNA expression was constant in all groups. Luteal cells treated with CM did not regulate mRNA levels of genes of steroidogenic enzymes, and cell survival genes. This data suggest CM from PA embryos do not regulate the transcription of genes associated with steroidogenesis and cell survival.

## Discussion

ISG15 is a protein expressed in the uterine endometrium on early pregnancy in response to the paracrine action of the embryo-derived IFNT in ruminants ( Austin *et al*., 2004 ). The presence of ISG15 protein and mRNA on the CL of Day 15 pregnant ewes suggests the possible endocrine action of IFNT ( Oliveira *et al*., 2008 ). The uterine vein infusion of IFNT extends luteal life span and protects the CL from PGF-induced luteolysis in ewes, further demonstrating the likely endocrine mechanism of action of IFNT on luteal tissue ( Bott *et al*., 2010 ; Antoniazzi *et al*., 2013 ). Luteal cells *ISG15* mRNA expression increased in response to recombinant ovine ( Antoniazzi *et al*., 2013 ) or bovine ( Shirasuna *et al*., 2015 ) IFNT in a dose-dependent manner, up to 1ng/ml. In our study, mixed luteal cell culture presented a dose-dependent response of ISG15 expression after the exposure to IFNA ( Fig. 2A ). The treatments of luteal cells with CM from embryos on Days 7, 9 and 12 had a similar pattern of expression of *ISG15* compared to those observed in the IFNA-treated cells. Based on that, our results reveal IFN-like response of luteal cells when exposed to CM from PA embryos, and this evidence was based on *ISG15* expression. Besides, it was also observed the longer the embryo develops *in vitro* the greater IFN activity is detected ( Figure 1 ). This suggests that the expression of *ISG15* in luteal cells is dependent on embryo-derived IFNs.

Parthenogenetic activation is used for research purposes to study several events at the moment of early embryonic development ( Soloy *et al*., 1997 ). On human embryos, parthenogenesis is a source of embryonic stem cells used as a model in several studies, due to religious, ethical, legal and political concerns that are raised when working with embryos capable of generating human life ( Brevini and Gandolfi, 2008 ). Parthenogenetic embryos are derived from a single egg developed without spermatic fertilization ( Henery and Kaufman, 1992 ). Oocytes in the metaphase 2 are activated by ionomicyn (mimicking the calcium wave induced by sperm penetration in the zona pelucida; Henery and Kaufman, 1992 ) followed by exposure to an actin polymerization inhibitor, usually cytochalasin B, originating diploid parthenotes ( Bałakier and Tarkowski, 1976 ). These PA embryos begin to express *IFNT* mRNA on the blastocyst phase at day 6 and the expression levels of *IFNT* mRNA was not different among *in vitro* fertilized (IVF), somatic cell nuclear transfer (NT) and PA embryos on Day 7 ( Yao *et al*., 2009 ). In our study, *IFNT* mRNA was not assessed. However, the antiviral assay detected Type I IFN activity in CM from Days 7, 9 and 12 of embryo development, with greater activity on Days 9 and 12. Furthermore, the mRNA expression of *ISG15* was not different on Days 7, 9 and 12, although these days were greater when compared to control group ( Fig. 2B ). The results presented herein suggest the Type I IFN activity detected on the antiviral assay may be from PA embryo-derived IFNT.

Interferon A and IFNT are Type I interferons, having similar three-dimensional structure ( Roberts *et al*., 1999 ) and analogous biological activities such as recognition of common classes of cell surface receptors ( Mogensen *et al*., 1999 ). The IFNA is more cytotoxic in Madin-Darby bovine kidney (MDBK) cell line than IFNT, but both have similar antiviral activity ( Subramaniam *et al*., 1995 ). Furthermore, IFNT was shown to be more antiluteolytic than IFNA, maintaining high plasma progesterone concentration for longer periods after estrus in ewes ( Green *et al*., 2005 ). Due to the similarity between IFNT and IFNA, and because the recombinant bovine IFNT is not yet commercially available, IFNA was used in our study as a positive control to validate the responsiveness of luteal cells based on their ability to express *ISG15* mRNA in response to Type I interferon. In another study, luteal cell culture treated with recombinant ovine IFNT reached the plateau of *ISG15* mRNA expression at 1 ng/ml ( Antoniazzi *et al*., 2013 ). However, when treated with IFNA, the plateau was reached at 10ng/ml, as expected, because IFNA effects on luteal cells are reduced when compared to IFNT ( Green *et al*., 2005 ).

Progesterone is a steroid hormone secreted by large and small luteal cells ( Niswender, 2002 ), which prevents luteolysis, regulates the growth and elongation of the blastocyst and the trophoblast secretion and maintenance of IFNT concentrations during the early pregnancy recognition and establishment ( Mann and Lamming, 2001 ). The steroidogenic enzymes cytochrome P450 cholesterol side chain cleavage (P-450scc/CYP11A1) converts cholesterol to pregnenolone ( Niswender, 2002 ), and then 3β-hydroxysteroid dehydrogenase (3β-HSD) converts pregnenolone to progesterone in the smooth endoplasmic reticulum ( Niswender, 2002 ). Luteal cell culture treated with 0.1 or 1ng/ml IFNT did not change progesterone secretion ( Shirasuna *et al*., 2015 ). Ewes infused with BSA or recombinant ovine (ro) IFNT did not alter progesterone concentrations in serum ( Antoniazzi *et al*., 2013 ). Both ovine IFNA and IFNT did not change plasma progesterone concentrations when infused in non-pregnant ewes in the same period ( Green *et al*., 2005 ). In our results, progesterone concentrations were not different in the culture medium of mixed luteal cells treated with CM or IFNA ( Tab. 2 ). Besides that, *CYP11A1* and *HSD3B1* mRNA expression were similar in all embryonic developmental days, in the different treatments with IFNA and the control. This confirms that neither CM nor IFNA stimulate progesterone secretion in luteal cells.

Bcl-2 family of proteins are involved in the regulation of apoptosis. Some members, as the Bcl-2, are antiapoptotic, while BAX is proapoptotic ( Adams and Cory, 1998 ). The serine/threonine kinase (AKT) or protein kinase B (PKB) contributes to the diverse cellular roles such as cell survival, growth, proliferation, angiogenesis, metabolism, and migration ( Manning and Cantley, 2007 ). XIAP (X-linked inhibitor of apoptosis) is a member of the inhibitor of apoptosis proteins (IAPs) family, and acts as an antiapoptotic molecules to inhibit the activity of some caspases, such as caspases 3 and caspases 7 ( Takahashi *et al*., 1998 ). Ewes infused with BSA and roIFNT did not present differences in luteal *AKT* mRNA expression, but increased *XIAP* mRNA in the roIFNT-infused group ( Antoniazzi *et al*., 2013 ). Luteal cell culture treated with CM on Days 7, 9 and 12 did not change *BAX* , *BCL2*, *AKT* and *XIAP* mRNA expression when compared to the control group ( Fig. 4C-F ). Luteal cell culture treated with IFNA did not change cell survival genes expression ( Fig. 3C-F ), demonstrating that IFNA does not regulate cell survival/apoptosis of *in vitro* cultured luteal cells.

In conclusion, findings of the present study support the hypothesis that luteal cell culture increase *ISG15* mRNA expression when treated with PA CM, which present Type I IFN activity. This antiviral activity present on CM from PA embryos may be from IFNT. Furthermore, luteal cell culture maintains progesterone secretion and no difference were observed on steroidogenic genes, suggesting the culture system was well established. Taken these together, CM from PA embryo culture present antiviral activity, luteal cells respond to Type I interferon and express ISG15. Finally, luteal cell culture is a valid system to evaluate Type I interferon response. These data indicate this model can be useful for IFNT endocrine signaling studies.
